# 

**Published:** 1865-07

**Authors:** 


Volume XXII. Number 7.

Dr. R. C. Hammill Box 130

### THE CHICAGO

### MEDICAL JOURNAL

De LASKIE MILLER, M. D.,

EPHRAIM INGALS, M. D.,

Editors and Proprietors.

JULY, 1865.

CHICAGO:
J. C. W. BAILEY, BOOK AND JOB PRINTER, 164 CLARK STREET.

1865.

